# Salutogenic Principles in Workplace Health Promotion Interventions: A Scoping Review

**DOI:** 10.1007/s10935-026-00915-9

**Published:** 2026-06-18

**Authors:** Sammy J. S. Wrede, Ines-Maria Gärtner, Achim Mortsiefer

**Affiliations:** 1 Chair of Occupational Medicine and Corporate Health Management, Department of Human Medicine, Faculty of Health, Witten/Herdecke University, Witten, Germany; 2 Institute of General Practice and Primary Care, Department of Human Medicine, Faculty of Health, Witten/Herdecke University, Witten, Germany

**Keywords:** Salutogenesis, Workplace health promotion, Occupational health, Empowerment, Participation, Sense of coherence, Behavioral interventions

## Abstract

While workplace health promotion has traditionally emphasized risk reduction, knowledge about the extent to which interventions incorporate health-promoting resources such as Sense of Coherence, Participation, Goal-Setting, Variability, and Empowerment is fragmented. This scoping review examined how these salutogenic principles are operationalized in behavioral workplace health promotion programs focused on primary prevention. A systematic search across five databases identified 128 intervention studies published between 2014 and 2024. Each program was assessed using a Salutogenesis-Score (0–5) reflecting the number of salutogenic principles integrated into its design and delivery. Overall, the incorporation of salutogenic principles was moderate. Empowerment, Variability, and Sense of Coherence were most frequently represented, whereas Participation and Goal-Setting appeared considerably less often. For instance, tailored interventions that included individualized counseling or self-management tools achieved high scores, with multi-component programs showing the strongest overall alignment. Although many programs contain elements that support health-promoting behavior, their potential to fully activate salutogenic mechanisms remains underused. Strengthening participatory approaches and personalized goal-setting may enhance employee agency, adaptability, and long-term engagement, thereby fostering more sustainable and empowering workplace health promotion.

**Supplementary Information:**

The online version contains supplementary material available at https://doi.org/10.1007/s10935-026-00915-9.

## Contribution to Health Promotion

- Explores how occupational health promoting programs strengthen individual health resources
- Identifies key factors like participation, personal goal setting, and flexibility of the program that support long-term health behavior
- Reveals that many programs still miss the chance to fully engage workers in meaningful ways
- Highlights the importance of empowerment and personalized support in workplace health promotion
- Offers inspiration for designing future programs that better match individual needs

**Supplementary Information:**

The online version contains supplementary material available at https://doi.org/10.1007/s10935-026-00915-9.

## Background

Workplaces are dynamic environments where health-promoting resources and health-compromising risks coexist. A recent *Lancet* series emphasizes that occupational health research has historically prioritized narrowly defined risks, thereby underestimating the broader role of work as a social determinant of health (The Lancet, 2023). Even in countries with robust healthcare systems such as Germany, prevention and health promotion remain under-resourced, with curative approaches continuing to dominate (Zeeb et al., 2025).

Health development can be advanced through both disease prevention by minimizing exposure to risk factors as well as health promotion by strengthening personal and contextual resources. Yet, academic and practical focus continues to be skewed toward pathogenic models of health, with comparatively limited attention given to salutogenic or resource-oriented frameworks (Eriksson & Lindström, 2005). Salutogenesis, as introduced by Antonovsky (1996), offers a complementary perspective by shifting focus from the causes of disease to the origins of health. This paradigm emphasizes the identification, mobilization, and reinforcement of internal and external health resources. This aligns with the Job Demands–Resources (JD-R) model, which emphasizes that job resources such as autonomy, social support, and learning opportunities can buffer job demands and enhance motivation and well-being (Bakker & Demerouti, 2007; Taris & Schaufeli, 2015). Accordingly, the World Health Organization (1986) defines health promotion as “the process of enabling people to increase control over, and to improve, their health.”

Central to Salutogenesis is the concept of (1) *Sense of Coherence (SoC)*, referring to a global orientation through which individuals perceive life as comprehensible, manageable, and meaningful (Antonovsky, 1996). In the workplace, SoC fosters resilience by enabling employees to understand their roles, mobilize resources, and derive purpose from their work (Bauer et al., 2015). (2) *Participation* emphasizes the active involvement of employees in the development and implementation of interventions. Participatory approaches enhance ownership, contextual fit, and long-term effectiveness by recognizing employees as co-creators rather than passive recipients (Campmans et al., 2022; Paterson et al., 2024). (3) *Goal-setting* is a motivational mechanism that structures behavior by directing attention, sustaining effort, and fostering feedback. Co-created and personalized goals enhance agency and align individual motivation with organizational values (Schunk & DiBenedetto, 2016). (4) *Variability* reflects the need for flexible, adaptive interventions that accommodate individual needs, preferences, and organizational settings. Rather than applying universal solutions, tailored programs are more effective in supporting self-regulation and behavioral change (Bergman et al., 2020; Faltermaier, 2017). (5) *Empowerment* underpins all other principles by promoting autonomy, competence, and social connectedness. It enables individuals to take control of their health through participatory decision-making, self-efficacy, and psychological safety (Holden et al., 2005; Wallerstein, 2006). For instance, Schopp et al. (2017) found that employees participating in a self-management program showed greater improvements in physical activity, vitality, and mental well-being compared to those in a control group.

Drawing on the multidimensional framework of “Positive Health”—which defines health as the ability to adapt and self-manage in the face of physical, emotional, and social challenges (Huber et al., 2011, 2016)—salutogenic principles provide a strong foundation for developing holistic, resource-oriented occupational health programs (OHPs). Although enabling conditions such as involving employees as stakeholders, securing leadership commitment, leveraging internal structures, and offering meaningful incentives have been outlined (Wrede et al., 2024), empirical insights into how salutogenic principles are actually translated into OHPs remains limited. A recent review by van Vliet et al. (2024) further indicates that the implementation of the Positive Health model in workplace settings remains scarce. Less is known about the concrete integration of salutogenic principles in OHPs. Despite the central role of Salutogenesis in health promotion training and discourse, the extent to which contemporary OHPs embody its principles has not yet been examined.

The present study addresses this gap by systematically reviewing how salutogenic principles are operationalized in behavioral workplace health promotion programs focused on primary prevention. Rather than merely mitigating health risks, these interventions aim to strengthen well-being through self-directed, participatory, and adaptive strategies. This scoping review investigates how salutogenic principles are embedded in intervention design and delivery across diverse workplace settings and intervention types. In doing so, it assesses whether OHPs enable employees to act and adapt in favor of their health.

## Method

To evaluate the salutogenic content of OHPs this scoping review was prepared according to the guidelines set out by the Joanna Briggs Institute in “Guidance for conducting systematic scoping reviews” (Peters et al., 2015) and its subsequent update “Updated methodological guidance for the conduct of scoping reviews” (Peters et al., 2020). Furthermore, the “Preferred Reporting Items for Systematic reviews and Meta-Analyses extension for Scoping Reviews (PRISMA-ScR) Checklist” (Tricco et al., 2018) and the flowchart proposed in “The PRISMA 2020 statement: an updated guideline for reporting systematic reviews” (Page et al., 2021) were used.

The PCC (population, concept, and context) framework has been applied to elaborate the research question (von Elm et al., 2019), followed by;

1. Development of the search strategy;
2. Specification of in-/exclusion criteria;
3. Abstract screening and study selection;
4. Data extraction;
5. Synthesis of findings.

From September to November, 2024, a systematic search was conducted using the electronic databases MEDLINE, CINAHL, APAPsycInfo, Psychology and Behavioral Science Collection via EBSCOhost, to identify research that explored workplace health promoting programs. The search was constructed based on the first strategy and further developed for appropriate search results without the application of additional filters. Searches were executed using keywords such as “workplace health promotion intervention”. Primary studies published in the last 10 years (2014—2024) available in English or German have been included. The search terms combined keywords related to the workplace (work*, occupation*, organi?ation*, employ*, job, staff), health promotion interventions (“health promotion*”, “health intervention*”, “health program*”, “health training*”), and study design (“original research”, “intervention* study”, “randomi?ed controlled trial”), which were connected using Boolean operators. Basic inclusion criteria were established and adapted as the research team became more familiar with the literature, so that they were applied to the totality of results obtained (Arksey & O'Malley, 2005).

Eligible studies targeted working populations and included primary prevention, behavioral interventions. Accordingly, studies were excluded if they addressed non-working populations (such as retirees, students, or children), clinical or community-based contexts, or participants described as patients. Further exclusions applied to studies on secondary or tertiary prevention, purely condition-specific outcomes, or interventions lacking behavioral components, as well as environmental-only measures (e.g. sit-stand workstations without behavioral strategies). Study protocols, and feasibility or pre-intervention studies were also excluded. Articles were screened for sufficient details on the interventions to allow for the assessment of salutogenic principles; lack of such detail led to exclusion. The first selection of articles via abstracts was performed in Rayyan “Software as a Service” (Ouzzani et al., 2016). Due to a sufficient number of studies no articles were retrieved by hand search.

Full-text analysis has been realized in citavi 6 as a tool to organize and review the articles. The selection of the final database of articles was made according to the criteria proposed. Subsequently, data extraction was performed by adjusting the fields proposed by the Joanna Briggs Institute in “Guidance for conducting systematic scoping reviews” (Peters et al., 2015) for the studies’ purposes: *type of the intervention (mental health, physical activity, consumption behavior, multi-component), authors and year of publication, population and sample, name of the intervention, duration of the intervention, main results, intervention details* (see APPENDIX).

To approach the research objective, salutogenic principles have been qualitatively derived for which the intervention details were then investigated. The following criteria that arose from the literature about Salutogenesis have been pretested on some intervention studies and accordingly defined as in the table below (Table 1).

**Table 1:** Definition of the salutogenic principles

No	Salutogenic principle	Yes (1) vs. No (0)	
	Sense of Coherence (SoC)	Aiming for wellbeing beyond risk minimization	Reduction of disease risks only
	Participation in the development of the intervention (P)	Staff was involved in invention	Dictated by management
	Individual Motivation/ Goal-Setting (GS)	Personal goals	No or rigid goals
	Individual Variability of the intervention (V)	Individually adaptable or coaching/counseling included	Pre-set program “join in or leave it”
	Empowerment (E)	Self-management (component)	No control or by others

*SoC* has been rated, if the intervention addresses the wellbeing of workers and was not perceived as merely prevention, i.e. risk minimization for the employer, but promotes health in a more holistic understanding including non-occupational health. *Participation* (P) is applied in “tailored” programs, when employees or representatives are involved in the implementation of the intervention before the training starts (e.g. intervention chosen based on employee surveys or supervisors take part in intervention planning). If goals are set individually or employees get the chance to define personal goals, individual motivation is given and rated as *Goal-Setting* (GS). When workers have the possibility to adapt the program to their own needs during the training or different options are offered (e.g. option to choose between program components or self-reflection anchored within the training to transfer the lessons learned to their own life or a coaching/counseling component), the intervention includes *Variability* (V). In this study *Empowerment* (E) is meant by enhanced self-management by the program, such as materials (e.g. booklets or audio discs), provided environmental modifications like sports equipment or trackers (e.g. via app or pedometer) to integrate the healthy behavior outside of and beyond the training.

In order to classify the salutogenic content and compare the interventions by category, i.e. “type of intervention”, a “Salutogenesis-Score” (0—5) has been build. This constructive part of the study is to be understood as exploratory. Classification of the Salutogenesis-Score according to the number of salutogenic principles were coded for the intervention as followed:

- 0—1 = Low
- 2—3 = Moderate
- 4—5 = High

All authors contributed to the qualitative rating process. AM and SJSW primarily developed the conceptual framework, while IMG and SJSW were responsible for conducting the ratings. The initial assessments were carried out independently by the researchers. Subsequently, the ratings were compared. In cases of discrepancies regarding the assignment of salutogenic principles to each OHP, the researchers engaged in discussion until consensus was reached. These discussions also facilitated refinement of the criteria and led to a more precise differentiation through an iterative process. The involvement of at least two researchers in each assessment ensured interrater reliability.

## Results

**Fig. 1:**
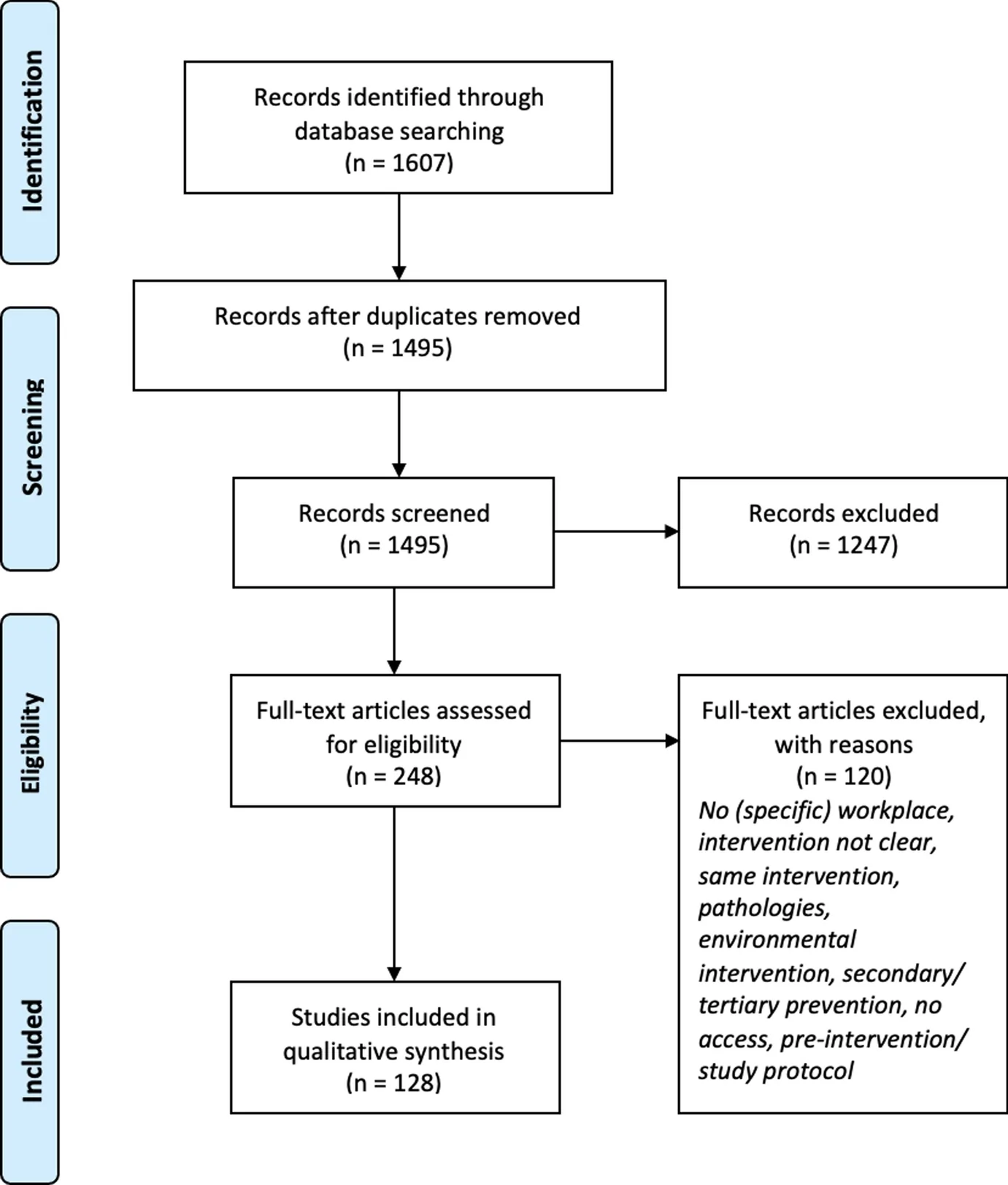
Screening process

The initial database search yielded a total of 1607 records, of which 112 duplicates were removed. The remaining 1495 records were screened based on titles and abstracts. Subsequently, 248 full-text articles were assessed for eligibility. Of these, 120 articles were excluded for reasons such as a focus on secondary or tertiary prevention (see Fig. 1). Identical interventions were only included if implemented in distinct organizational contexts. Studies initiated outside the workplace, such as those led by insurance providers without integration into organizational processes, were excluded. In several cases, studies were excluded due to insufficient documentation of intervention content, which impeded the assessment of underlying salutogenic mechanisms. Ultimately, 128 studies met all inclusion criteria and were included in the final synthesis of this scoping review. The final sample included primarily studies on behavioral OHPs, in accordance with the inclusion criteria outlined in the methods section. Table 2 beholds all included studies divided into category and differentiated by Salutogenesis-Score (for a table including more details, the coding for salutogenic principles and Salutogenesis-Score per intervention see APPENDIX).

**Table 2:** Included studies by salutogenic content

Level of salutogenetic content		Low	Moderate	High
Type of intervention	N	0—1	2—3	4—5
Mental health	25		Coelhoso et al. (2019); Gayed et al. (2019); Gayed et al. (2024); Grabbe et al. (2020); Hirshberg et al. (2022); Hsu et al. (2021); Huang et al. (2015); Imamura et al. (2014); Jahnke et al. (2023); Lee (2023); Milligan-Saville et al. (2017); Milner et al. (2020); Miquel et al. (2023); Sasaki et al. (2021); Thielecke et al. (2024); Thiart et al. (2015); Trombka et al. (2021); Umanodan et al. (2014); Watanabe et al. (2019); Watanabe et al. (2024)	Bostock et al. (2016); Darviri et al. (2023); Janssen et al. (2023); Mediavilla et al. (2023); van Berkel et al. (2014)
Physical activity	49	Barene et al. (2014); Calogiuri et al. (2015); Chandrasekaran et al. (2024); Hamilton et al. (2019); Laal et al. (2017); Li et al. (2017); Maddux et al. (2018); Mainsbridge et al. (2014); Mayer et al. (2015); Oka et al. (2019); Patel et al. (2016); Rasotto et al. (2015); Santos et al. (2020)	Akyurek et al. (2022); Alexander et al. (2015); Alexopoulos et al. (2014); Andersen et al. (2015); Bale et al. (2015); Berninger et al. (2022); Boerema et al. (2019); Cocker et al. (2017); Dalager et al. (2016); Danquah et al. (2017); Finkelstein et al. (2016); Fritz Lerche et al. (2024); Gu et al. (2020); Hadgraft et al. (2017); Hunter et al. (2018); Kajiki et al. (2017); Lidegaard et al. (2018); Lithopoulos et al. (2020); Mansi et al. (2015); Matsugaki et al. (2017); Maylor et al. (2018); Miranda Bispo et al. (2020); Murray et al. (2019); Nooijen et al. (2020); Ojo et al. (2024); Pedersen et al. (2019); Peterman et al. (2019); Raedeke & Dlugonski (2017); Renaud et al., (2020); Rollo & Prapavessis (2020); Taulaniemi et al. (2019); Ting et al. (2019); Vining et al. (2020)	Dadaczynski et al. (2017); Ghaffari et al. (2024); Park & Hwang (2024)
Consumption behavior	10	van den Brand et al. (2018); Watanabe et al. (2018)	Fitzgerald et al. (2020); Pidd et al. (2018); Shrestha et al. (2024); Thorndike et al. (2016)	Agarwal et al. (2015); Asfar et al. (2021); Meyn et al. (2022); Rameshbabu et al. (2018)
Multi-component	44	Kullgren et al. (2016); Reif et al. (2020); Taylor et al. (2016); van Holland et al., (2018)	Angerer et al. (2015); Das Gecim et al. (2021); Gupta et al. (2018); Hoosen et al. (2024); Kempf et al. (2019); Kim et al. (2024); Linnan et al. (2020); Patel et al. (2018); Ritngam et al. (2024); Roos et al. (2024); Roussel et al. (2015); Sampson et al. (2019); Soler-Font et al. (2019); Suni et al. (2018); Thorndike et al. (2014); van Schaaijk et al. (2019); Watanabe & Kawakami (2018); Zoellner et al. (20160	Addley et al. (2014); Aegerter et al. (2023); Balk-Møller et al. (2017); Clemes et al. (2022); Coffeng et al. (2014); Cook et al. (2015); Das et al. (2019); Day et al. (2019); Hammer et al. (2019); Hammer et al. (2021); Lippke et al. (2015); Lopes et al., (2023); Miller et al. (2016); Oftedal et al. (2019); Olson et al. (2016); Olson et al.^2016^ Peters et al. (2018); Puhkala et al. (2015); Smit et al. (2024); Viester et al. (2018); Weinhold et al. (2015); Wilson et al. (2016)

More details of the included studies are provided within the APPENDIX

Table 3 displays the results on a meta-perspective, which will be discussed for each category in the subsequent sections.

**Table 3:** Numerical and percentual results by type of intervention, salutogenic principles, and mean “Salutogenesis-Score”

Type of intervention	N	SoC	P	GS	V	E	Salutogenesis-Score (mean)
Mental health	25	0.92	0.28	0.16	0.76	0.76	2.9
Physical activity	49	0.35	0.24	0.29	0.53	0.82	2.2
Consumption behavior	10	0.90	0.30	0.20	0.70	0.50	2.6
Multi-component	44	0.80	0.23	0.52	0.82	0.86	3.2
Total	128	0.74	0.26	0.29	0.70	0.74	2.7

*SoC* sense of coherence, *P* participation, *GS* goal-setting, *V* variability, *E* empowerment; percentual values represent the proportion of interventions within each category that were coded for the respective salutogenic principle and mean “Salutogenesis-Score” indicates the average number of salutogenic principles identified per intervention within a category (range 0–5)

In the following, the meta-results per category are presented as well as some example interventions with high Salutogenesis-Scores as defined in the methods section. Within the presentation of the exemplary studies, the coded salutogenic principles are marked in square brackets behind the mentioned components that led to coding.

### Mental Health

25 mental health interventions were included in this study. On average, these interventions get a Salutogenesis-Score of 2.8 (Table 3), which corresponds to a moderate score based on the criteria defined in the methods section. Among the coded salutogenic principles, *Sense of Coherence* (0.92) was the most frequently addressed, while *Goal-Setting* (0.16) and *Participation* (0.28) were rated considerably less often.

One of the highest-rated interventions (Salutogenesis-Score: 4) in this category is a digital cognitive behavioral therapy program for insomnia, which significantly improved sleep outcomes among British office workers over an 8-week period (Bostock et al., 2016). This intervention adopted a holistic health approach and featured an animated virtual therapist [SoC]. Personalization was achieved through algorithm-driven tailoring to individual characteristics [V], and participants set personal goals [GS]. The program also included progress tracking via a sleep diary [E], along with system-generated email/SMS prompts and access to a post-moderated online community.

A similarly high-rated intervention (Salutogenesis-Score: 4) is the “Pythagorean Self-Awareness” program, which demonstrated significant improvements in stress, depressive symptoms, self-esteem, loneliness, self-efficacy, and anger among Greek civil servants over an 8-week period (Darviri et al., 2023). The intervention emphasized structured self-reflection based on the 71 Golden Verses of Pythagoras, encouraging emotional detachment and self-awareness [SoC]. Sessions were held weekly and lasted one to two hours, combining individualized diaphragmatic breathing training and psychoeducation on stress, health behaviors, and lifestyle choices. Personal goal setting was integrated into the program, requiring participants to reflect and define actionable personal objectives [GS]. Engagement was supported by daily self-practice, which involved two 30-min sessions in a quiet environment following breathing exercises [V]. Participants received printed materials for guidance, including step-by-step instructions for self-awareness exercises, promoting continuity and internalization of new habits [E].

Another high-scoring example with a Salutogenesis-Score of 4 is the “Mindful VIP” program, evaluated across multiple studies and shown to enhance work engagement among Dutch researchers over an 8-week period (van Berkel et al., 2014; van Dongen et al., 2016). This intervention combined mindfulness training [SoC] and e-coaching [V] with supportive environmental components such as fruit and vegetable offerings, lunch walking routes, and a buddy system. Participants developed personal energy plans by setting their own goals [GS], responding to the guiding question: “What do I need to do to feel well at work?” Although sessions were held onsite over eight weeks, they occurred outside of working hours. Additional materials included handouts with homework assignments, a mindfulness exercise booklet, and audio discs for relaxation exercises [E]. The buddy system also supported peer exchange, while a dedicated intranet page offered local walking route suggestions.

### Physical Activity; Relaxation, Exercise, and Ergonomics

49 interventions related to physical activity were included in this study, comprising exercise (n = 30), ergonomics (n = 13), and relaxation (n = 6). On average, physical activity interventions get a moderate Salutogenesis-Score of 2.2 (Table 3). Among the salutogenic principles, *Empowerment* was most frequently coded (0.8), while *Sense of Coherence* (0.33), *Participation* (0.27), and *Goal-Setting* (0.29) were less commonly addressed in this category.

One exercise intervention with a high Salutogenesis-Score (4) was “Healingo Fit,” a 6-week, browser-based program implemented among German automobile manufacturing workers (Dadaczynski et al., 2017). Aiming to increase physical activity, the program used pedometers and digital tools as part of a universal preventive approach [E]. It incorporated evidence-based behavior change methods targeting knowledge (e.g., information provision), intention (e.g., goal setting), volition (e.g., action planning), self-efficacy (e.g., self-monitoring and feedback), and social norms (e.g., encouragement, social comparison) [SoC]. Gamified elements such as achievement badges and daily step targets were delivered via email. The intervention comprised four modules: individualized step goals [GS], lifestyle quizzes, the selection of general health goals [V], and participation in team or individual challenges. Walking time as well as health promotions outcomes such as knowledge, intention, and self-efficacy increased due to the intervention.

Another intervention with a Salutogenesis-Score of 4 was based on the Transtheoretical Model and targeted female healthcare workers in Iran (Ghaffari et al., 2024). Participants were first categorized into five stages of change (pre-contemplation, contemplation, preparation, action, maintenance) based on pre-tests, followed by a needs analysis to tailor educational materials [P]. Over one month, educational sessions were held in urban healthcare centers or bases, featuring lectures and presentations approved by the Ministry of Health [SoC]. Materials included pamphlets, slides, posters, and CDs [E], and were adapted to participant comprehension levels. Each session included group discussions and the definition of personal behavioral goals [GS]. The intervention managed to increase engagement in physical activity.

An intervention with the highest Salutogenesis-Score (5) was a 12-week digital physical activity promotion program for male office workers in South Korea, which successfully increased basic psychological needs, intrinsic motivation, health promotion behavior, and physical and physiological indicators (Park & Hwang, 2024). Delivered via Zoom, the program began with individual interviews to assess needs and barriers [P]. Weekly meetings alternated between health education and exercise sessions in odd weeks, and small group discussions with tailored counseling in even weeks [V]. Exercise videos, expert input, breakout sessions for peer exchange including personal lifestyle and goals [GS], and individualized motivational text messages supported behavior change [E]. The program emphasized intrinsic motivation and was continuously revised based on expert feedback and participant responses (e.g., simplified terminology, visual materials), fostering autonomy and relatedness through small group interactions [SoC].

A mid-range example (Salutogenesis-Score: 3) is the multi-component “Dynamic Work” intervention conducted among office workers in the Netherlands, aimed at reducing sitting time (Renaud et al., 2020). The intervention included environmental (e.g., sit-stand desks, desk bikes, stability balls), organizational (e.g., team sessions), and individual components (e.g., activity and sitting trackers) [E]. A face-to-face introductory meeting with an occupational physiotherapist introduced motivational strategies, including managerial role-modeling. Two group meetings provided education on sedentary health risks and ergonomic furniture use, and discussed individual experiences with the intervention. A tracking device offered real-time feedback on step counts, upright time, and sitting duration, with optional haptic feedback for prolonged sitting. A 12-week self-help booklet (“Sit Less, Move More”) supported individual goal setting [GS], and open consultations with occupational physiotherapists were available for further guidance [V]. The intervention increased occupational steps among staff.

### Consumption Behavior; Diet and Addictive Behavior

Due to the limited availability of data on diet (n = 7) and addictive behavior (n = 3) interventions, these categories were combined into a single category termed consumption behavior. In total, 10 interventions were included in this group. On average, consumption behavior interventions get a moderate Salutogenesis-Score of 2.6 (Table 3). Among the salutogenic principles, *Sense of Coherence* (0.9) and *Variability* (0.7) were most frequently coded, whereas *Participation* (0.3) and *Goal-Setting* (0.2) were the least represented.

A diet-focused intervention with a high Salutogenesis-Score (4) was conducted as an educational workplace health promotion program among German office workers in an offsite, full-time setting at a wellness hotel over three weeks and eventually managed to improve food literacy and dietary intake (Meyn et al., 2022). The intervention aimed to enhance health literacy and support self-regulation through cognitive and behavioral skill development [SoC]. Each participant underwent a comprehensive medical examination to determine baseline health status [V], followed by the formulation of individualized health goals in collaboration with health professionals. This participatory process was supported by motivational interviewing techniques [GS]. Program components included daily morning activation routines, structured group sessions, and one-on-one coaching. Educational content addressed nutrient composition and its physiological implications, portion control, caloric needs, and practical cooking skills. Topics such as the roles of macronutrients, associated health risks, behavior change strategies, and national dietary guidelines were conveyed through the use of nutrient tables and recipe-based learning resources [E].

Another highly rated intervention (Salutogenesis-Score: 5) targeted smoking cessation among U.S. construction workers and was tailored to the needs of a diverse workforce (Asfar et al., 2021). The intervention applied behavioral group counseling during lunch breaks near food trucks and was delivered in both English and Spanish to enhance accessibility and cultural relevance [SoC]. Personalization was achieved through feedback from Hispanic workers and coordination with healthcare staff and management, ensuring the program addressed specific workplace and community needs [V]. Goal setting was embedded in the quit planning process, with participants supported in establishing a quit date and developing relapse prevention strategies [GS]. Engagement tools included two brief follow-up phone calls around the quit date, a fax referral system to the tobacco quitline, and access to up to four phone counseling sessions as well as nicotine replacement therapy (NRT) for up to eight weeks [E]. Counseling content focused on quitting preparation, navigating initial withdrawal, long-term relapse prevention, and proper NRT use. The 8-week intervention, supported by a worksite safety manager [P], was effective in significantly improving abstinence rates among participants.

### Multi-Component Including Weight Management

44 multi-component (n = 30) and weight management (n = 14) interventions have been included in this study. The multi-component interventions get a Salutogenesis-Score of 3.2 on average (Table 3), which equals a moderate almost high score. Highest values are being noted for *Empowerment* (0.86), *Variability* (0.82), and *Sense of Coherence* (0.80), but the salutogenic content is high in general in the multi-component category. Only *Participation* (0.23) has not been rated much.

A 6-months structured health intervention for British truck drivers achieved a high Salutogenesis-Score (4) and led to favorable increases in mean daily steps, reduced sitting time, and improved engagement in moderate-to-vigorous physical activity (Clemes et al., 2022). The intervention, grounded in Social Cognitive Theory, was designed to modify behavior around physical activity, diet, and sedentary time, especially outside of driving hours [SoC]. It began with a 6-h, group-based education session delivered by trained peers (truck drivers acting as driver trainers), focusing on health risks associated with inactivity and poor diet [V]. Participants set individual goals and created action plans based on personalized baseline feedback [GS]. To support implementation, drivers received resources such as resistance bands, grip strength tools, and a self-monitoring device that gave real-time activity feedback [E]. The practical “cab workout” was introduced and practiced during the sessions.

Another highly rated intervention (Salutogenesis-Score: 5) was implemented over 12 months among Dutch financial service workers and resulted in reduced exhaustion, increased vigorous physical activity, and more frequent short breaks and active commuting (Coffeng et al., 2014). The intervention combined social and environmental strategies to enhance workplace vitality [SoC]. Behavioral change was encouraged through motivational interviewing delivered in small teams by team leaders trained by professionals [P]. During four 90-min group sessions over 3.5 months, participants articulated personal goals and identified rewards for goal achievement using structured worksheets [GS]. A web-based social platform further supported engagement [E]. Physical spaces were redesigned to promote health behaviors, with zones featuring standing meeting tables, informal activity spaces like table tennis, and ergonomic furniture integrated into daily work routines [V].

A 9-months workplace-based weight management intervention for Danish social and healthcare workers received a Salutogenesis-Score of 4 and resulted in significant reductions in body weight, body fat percentage, and waist circumference (Balk-Møller et al., 2017). The intervention promoted a healthy lifestyle through a web- and app-based platform that facilitated self-regulation and engagement [SoC]. Tailored feedback and gamification elements (like point rewards and team competitions) increased personal relevance [V]. Participants selected personal or suggested pledges and received goal-specific motivational messages [GS]. The program offered social incentives (e.g., group activities), visual progress feedback, and video-based exercise routines. Additional features included tools for diet tracking, smoking cessation, and digital resources accessible via the online portal [E].

An occupationally tailored, web-based intervention for U.S. firefighters (Salutogenesis-Score: 4) demonstrated positive effects on weight loss over 6 months (Day et al., 2019). This digital program focused on improving nutrition, physical activity, and behavioral health in a work-relevant context [SoC]. Weekly educational emails addressed hydration, diet quality, and reducing processed food consumption, all tailored to firefighter routines. Participants set personal health goals and action plans, supported by behavior-tracking tools and feedback mechanisms [GS]. The program featured structured 6-week exercise routines at three skill levels, with video/audio demonstrations suitable for gyms, fire stations, or home [E]. Optional telephone coaching with motivational interviewing was available but used sparingly, as the intervention was intentionally low-intensity [V].

## Discussion

This scoping review synthesized 128 OHPs, offering a comprehensive overview of how salutogenic principles are currently integrated into practice. Overall, mean Salutogenesis-Scores across categories were moderate, with multi-component programs showing the strongest alignment. By contrast, physical activity interventions displayed the greatest potential for improvement. Most studies were conducted in office settings across European countries and primarily focused on behavioral-level interventions.

Across the five evaluated salutogenic principles, *Empowerment* and *Variability* were moderately reflected, while *Participation* and *Goal-setting* remained underutilized. *SoC* presented a heterogeneous pattern: higher alignment was found in mental health, consumption behavior, and multi-component interventions, while physical activity programs performed less favorably. These disparities likely reflect differences in conceptual scope—whereas mental health and consumption-related programs often address diffuse boundaries between work-related and personal factors, physical activity interventions are typically more risk-compensatory and limited in breadth. Multi-component programs inherently adopt a broader perspective and thus more frequently incorporate multiple salutogenic dimensions.

To enhance salutogenic quality, future OHPs should place greater emphasis on participatory processes and personal goal-setting. These components are critical for strengthening perceived autonomy, motivation, and individual agency. Mental health interventions perform above average, but individual goal-setting is comparatively absent—this may be partly due to the non-directive character of mindfulness-based approaches (Kabat-Zinn, 2003). Yet, recent findings show that integrating participatory and personalized elements from the outset can improve program engagement and outcomes, particularly in heterogeneous workforces (Campmans et al., 2022; Paterson et al., 2024). Narrative-based and tailored interventions—particularly in consumption-related behaviors—have proven effective in secondary prevention. For example, digital storytelling has shown long-term benefits in health literacy and engagement (Shannon & Parker, 2022). Tailoring strategies also help reach vulnerable or high-stress groups, especially as part of broader communication frameworks (Jensen et al., 2014; Oftedal et al., 2019).

Digital interventions were prominently featured and demonstrated high potential to scale personalized health promotion through tools such as gamification, feedback, and mobile access. These approaches reduce access barriers and can effectively engage new target groups (Peláez Zuberbuhler et al., 2025). Evidence supports their efficacy across domains such as physical activity (Moreira et al., 2022), mental health (Paterson et al., 2024), and ergonomic education (Kazemi et al., 2022). Nevertheless, concerns about digital literacy, equity, and long-term sustainability remain. One innovative example involved the use of a social robot to guide employees through interactive sessions combining self-efficacy training, goal-setting, and digital monitoring—achieving high salutogenic alignment (Lopes et al., 2023). However, performance and sustainability comparisons between high-tech and high-touch interventions should be carefully considered (Edmunds et al., 2021).

Despite considerable variability in intervention duration and intensity—from short digital modules to year-long, in-person programs—positive effects were observed across all categories in areas such as physical activity, nutrition, sleep, and mental wellbeing. Interestingly, shorter, well-designed interventions (e.g., Day et al., 2019) sometimes outperformed longer programs (e.g., van Holland et al., 2018), underlining the importance of conceptual quality over intervention length. Differences in Salutogenesis-Scores even among similar interventions (e.g., ergonomic programs) further support this conclusion. However, inconsistencies in outcome reporting limit the generalizability of these findings.

A key inclusion criterion was the organizational integration of interventions. Programs without workplace anchoring (e.g. via external providers) were excluded to maintain ecological validity and contextual coherence—core aspects of salutogenic theory. These findings align with integrated occupational health models (Punnett et al., 2020), which emphasize the importance of participatory governance, cross-sector collaboration, and systemic design in sustainable workplace health strategies.

In a nutshell, the results highlight the need for a paradigm shift: from a risk-focused model toward a systemic public health approach grounded in Salutogenesis. This includes addressing upstream determinants of health, fostering participation and empowerment, and creating enabling environments within organizations (Pega et al., 2023). Workplace health promotion must be embedded in interdisciplinary structures connecting occupational health, primary care, and public health systems (Robert Bosch Stiftung, 2021).

A particularly promising framework for such integration is the concept of “Positive Health”, developed in the Netherlands. Drawing on salutogenic theory, positive psychology, and lifestyle medicine, it redefines health as the ability to adapt and self-manage in the face of challenges (Bodryzlova & Moullec, 2023; Huber et al., 2011, 2016). This review demonstrates how salutogenic principles operationalize Positive Health in workplace contexts by shifting the focus from risk avoidance to the activation of individual and contextual resources. Interventions that emphasize adaptability, cultural relevance, and employee participation are best positioned to achieve lasting behavioral change.

### Limitations

The Salutogenesis-Score developed in this review is exploratory and does not indicate overall program quality or effectiveness. Scores reflect the presence or absence of defined salutogenic criteria in published descriptions, which may not fully capture real-world practices. Many effective interventions may have lacked detailed reporting, leading to lower scores despite likely salutogenic value in implementation. Moreover, contextual factors such as leadership support and organizational culture—known to influence health promotion success—were not systematically assessed (Wrede et al., 2024). While the origin of the interventions (e.g., workplace-driven vs. researcher-led) was not formally coded, externally developed programs may show weaker alignment with organizational context.

A key limitation lies in the reliance on published reports. Only explicitly documented content was evaluated; no assumptions were made regarding undocumented program features. This conservative approach supports transparency and replicability but may underestimate the actual presence of salutogenic principles. Several relevant studies were excluded due to insufficient reporting on intervention design or theoretical underpinnings, highlighting the need for more consistent and detailed publication standards.

To maintain conceptual clarity, only intervention study designs were included, excluding exploratory or formative studies that may offer valuable insights into intervention development. This focus narrows generalizability, particularly for systemic interventions or small enterprise initiatives where salutogenic elements may be present informally but go undocumented.

Health outcomes were not systematically analyzed, limiting conclusions on the relationship between Salutogenesis-Scores and intervention effectiveness. Standardized reporting of outcomes would enhance comparability and impact assessment.

Lastly, evaluating salutogenic principles required qualitative interpretation. Differentiating constructs such as *Participation* and *Variability* was not always straightforward. For example, assessments were only coded as *Participation* if conducted prior to implementation and involving employees. Assessments used solely for evaluation or research purposes were excluded unless they informed program modification (then coded as *Variability*). *SoC*, in particular, required holistic judgment rather than keyword-based coding. To enhance reliability, a pretest and interrater checks were conducted.

Future research—especially meta-analyses and original studies—should further refine salutogenic evaluation frameworks, improve reporting standards, and test the assumptions underlying this assessment model.

## Conclusion

This study examined whether workplace health promoting programs empower employees to behave healthily, act, and adapt in favor of their health, using salutogenic principles as an analytical framework. Across all categories, the average salutogenic content was moderate, with multi-component interventions achieving the highest values. These programs often integrated behavioral, environmental, and organizational strategies, thereby addressing multiple salutogenic dimensions simultaneously. The findings suggest that while principles like *Empowerment*, *Variability*, and *Sense of Coherence* were commonly reflected, *Goal-Setting* and especially *Participation* were notably underrepresented. This imbalance points to a broader challenge in workplace health promotion: although interventions are increasingly evidence-based and technically sophisticated, they often lack meaningful employee involvement in their design and implementation despite the centrality of participatory approaches in salutogenic frameworks.

Regarding the core research question, the results show that many interventions provide a foundation for health-promoting behavior, but do not consistently empower workers in a salutogenic sense. Empowerment in its complete understanding requires not only offering health-related information or tools but also actively involving employees in shaping the interventions that affect their wellbeing. Programs need to move beyond risk prevention toward activating, dialogical, and context-sensitive approaches that foster individual agency and collective ownership*.* Workplace health promotion may benefit from promising salutogenic interventions like “Positive Health”. In conclusion, applying salutogenic principles more holistically can enhance both the acceptance and the impact of workplace health programs, guiding a shift from instructive to empowering health promotion.

## Supplementary Information

Below is the link to the electronic supplementary material.

Supplementary file1 (PDF 674 KB)
