# Supplementary material for: Salutogenic Principles in Workplace Health Promotion Interventions: A Scoping Review
Source: J Prev (2022). 2026 Jun 18;47(5):851–78. doi: 10.1007/s10935-026-00915-9 (PMC13582129; doi:10.1007/s10935-026-00915-9)
Supplement: Supplementary file 1 [file 10935_2026_915_MOESM1_ESM.pdf]

Appendix

Authors and year of publication	Population and sample	Name of the intervention	Duration of the intervention	Type of the intervention	SoC	P	GS	V	E	Salutogenesis-Score
Addley et al. 2014	United Kingdom; civil service workers	Workplace-based health risk appraisal intervention	12 months	Multicomponent	1	0	1	1	1	**4**
Aegerter et al. 2023	Switzerland; office workers	Multi-component intervention (NEXpro)	12 weeks	Multicomponent	1	1	1	0	1	**4**
Agarwal et al. 2015	United States; insurance workers	Nutrition intervention program	18 weeks	Diet	1	1	0	1	1	**4**
Akyurek et al. 2022	Turkey; nurses	Workplace health promotion program	5 weeks	Relaxation	1	0	0	0	1	**2**
Alexander et al. 2015	United States; nurses	Yoga	8 weeks	Relaxation	1	0	0	0	1	**2**
Alexopoulos et al. 2014	Greece; predominantly office workers	Relaxation program	8 weeks	Relaxation	1	0	0	0	1	**2**
Andersen et al. 2015	Denmark; healthcare workers	Physical exercise	10 weeks	Exercise	1	0	0	1	1	**3**
Angerer et al. 2015	Germany; apprentices	"Fit4You"- a programme for prevention and reduction of overweight in apprentices	2 years	Weight management	1	0	0	1	1	**3**
Asfar et al. 2021	United States; construction workers	Novel workplace smoking cessation intervention	8 weeks	Addictive behavior	1	1	1	1	1	**5**
Bale et al. 2015	United States; university workers	Using Time at Work to Exercise	9 months	Exercise	1	0	0	1	1	**3**
Balk-Møller et al. 2017	Denmark; social and healthcare workers	Web- and app-based workplace intervention to promote healthy lifestyle and weight loss “SoSu-life”	9 months	Weight management	1	0	1	1	1	**4**
Barene et al. 2014	Denmark; female hospital workers	Zumba	40 weeks	Exercise	1	0	0	0	0	**1**
Berninger et al. 2022	Netherlands; office workers	UPcomplish	12 weeks	Ergonomics	0	0	1	1	1	**3**
Boerema et al. 2019	Netherlands; office workers	mHealth intervention to reduce sedentary behaviour "PEARL app"	2 weeks	Ergonomics	0	0	0	1	1	**2**
Bostock et al. 2016	United Kingdom; predominantly office workers	Digital cognitive behavioral therapy for insomnia	8 weeks	Mental health	1	0	1	1	1	**4**

Calogiuri et al. 2015	Norway; office workers	Green exercise	2 weeks	Exercise	1	0	0	0	0	**1**
Chandrasekaran et al. 2024	India; office workers	SMART STEP	24 weeks	Ergonomics	0	0	0	0	1	**1**
Clemes et al. 2022	United Kingdom; truck drivers	Structured health intervention for truckers	6 months	Multicomponent	1	0	1	1	1	**4**
Cocker et al. 2017	Belgium; office workers	Start to Stand	1 month	Ergonomics	0	0	1	1	0	**2**
Coelhoso et al. 2019	Brazil; female hospital workers	Mental health mobile app for well-being and stress reduction	8 weeks	Mental health	1	0	0	0	1	**2**
Coffeng et al. 2014	Netherlands; financial service workers	Worksite social & physical environment intervention	12 months	Multicomponent	1	1	1	1	1	**5**
Cook et al. 2015	United States; information technology workers	HealthyPast50	3 months	Multicomponent	1	1	0	1	1	**4**
Dadaczynski et al. 2017	Germany; automobile manufacturing workers	Healingo Fit	6 weeks	Exercise	1	0	1	1	1	**4**
Dalager et al. 2016	Denmark; office workers	Intelligent physical exercise training	1-2 years	Exercise	1	1	0	1	0	**3**
Danquah et al. 2017	Denmark; office workers	Take a Stand!-a multi-component intervention aimed at reducing sitting time	1-3 month	Ergonomics	0	1	1	0	1	**3**
Darviri et al. 2023	Greece; civil servants	Pythagorean self-awareness intervention	8 weeks	Mental health	1	0	1	1	1	**4**
Das et al. 2019	United States; (university) workers	Energy management training course	2.5 days	Multicomponent	1	0	1	1	1	**4**
Das Gecim et al. 2021	Turkey; nurses	Health training, yoga training and individual nutrition counselling	4-8 weeks	Multicomponent	1	0	0	1	1	**3**
Day et al. 2019	United Sates; firefighters	Occupationally tailored, web-based, nutrition and physical activity program	6 months	Weight management	1	0	1	1	1	**4**
Finkelstein et al. 2016	United States; predominantly office workers	Activity trackers with and without incentives to increase physical activity (TRIPPA)	6 months	Exercise	0	0	0	1	1	**2**
Fitzgerald et al. 2020	Ireland; manufacturing workers	Complex workplace dietary intervention	7-9 months	Diet	1	0	0	1	0	**2**

Fritz Lerche et al. 2024	Denmark; industrial workers	Goldilocks work intervention	12 weeks	Ergonomics	0	0	1	1	1	**3**
Gayed et al. 2019	Australia; managers	"HeadCoach" online mental health training	6 weeks	Mental health	1	0	0	1	0	**2**
Gayed et al. 2024	Australia and New Zealand; physicians	"HeadCoach" online mental health training	3 months	Mental health	0	1	0	1	1	**3**
Ghaffari et al. 2024	Iran; female healthcare workers	Intervention based on the transtheoretical model aimed at increasing physical activity	3-6 months	Exercise	1	1	1	0	1	**4**
Grabbe et al. 2020	United States; nurses	Community resiliency model	1 year	Mental health	1	0	0	1	1	**3**
Gu et al. 2020	China; workers	Group-based intervention program on physical activity	100 days	Exercise	0	1	0	0	1	**2**
Gupta et al. 2018	Denmark; industrial workers	Participatory physical and psychosocial workplace intervention	8-12 months	Multicomponent	0	1	0	1	1	**3**
Hadgraft et al. 2017	Australia; office workers	Stand Up Victoria	12 months	Ergonomics	0	1	0	1	1	**3**
Hamilton et al. 2019	Australia; office workers	10 top tips (10TT)	6 weeks	Exercise	0	0	0	0	1	**1**
Hammer et al. 2019	United States; construction workers	Safety and health improvement program	30-60-90 days	Multicomponent	1	1	1	0	1	**4**
Hammer et al. 2021	United States; army workers	Total Worker Health ® leadership intervention	5 weeks	Multicomponent	1	0	1	1	1	**4**
Hirshberg et al. 2022	United States; predominantly teachers	Healthy minds program	12 months	Mental health	1	0	0	1	1	**3**
Hoosen et al. 2024	South Africa; school workers	Health4LIFE weight loss intervention	16 weeks	Weight management	1	0	0	0	1	**2**
Hsu et al. 2021	Taiwan; healthcare workers	Zentangle art	9 months	Mental health	1	0	0	0	1	**2**
Huang et al. 2015	Taiwan; manufacturing workers	Mindfulness-based program	8 weeks	Mental health	1	0	0	1	1	**3**
Hunter et al. 2018	Australia; university workers	Moderate- to vigorous-intensity aerobic and resistance exercise program	8 weeks	Exercise	0	0	0	1	1	**2**

Imamura et al. 2014	Japan; information terchnology workers	Internet-based computerized cognitive behavior therapy	6 weeks	Mental health	1	0	0	1	1	**3**
Jahnke et al. 2023	United States; firefighters	Stress first aid	1 year	Mental health	0	1	0	1	0	**2**
Janssen et al. 2023	Netherlands; teachers	Mindfulness-based stress reduction	8 weeks	Mental health	1	1	0	1	1	**4**
Kajiki et al. 2017	Japan; managers	Ergonomic low back pain training	10 months	Ergonomics	0	1	0	1	1	**3**
Kempf et al. 2019	Germany; pharmaceutical workers	Telemedical coaching for weight loss	12 months	Weight management	0	0	0	1	1	**2**
Kim et al. 2024	Japan; information technology (remote) workers	Multicomponent intervention (individual, sociocultural, organizational)	8 weeks	Multicomponent	0	0	1	1	1	**3**
Kullgren et al. 2016	United States; healthcare workers	Employer matching of employees' monetary contributions to deposit contracts to promote weight loss	24-36 weeks	Weight management	0	0	0	0	1	**1**
Laal et al. 2017	Iran; tailors	Ergonomic intervention	3 months	Ergonomics	0	0	0	0	1	**1**
Lee 2023	South Korea; predominantly office workers	Mobile app-based stress management intervention	6 weeks	Mental health	1	0	0	1	1	**3**
Li et al. 2017	Australia; office workers	Sit-stand protocols	4 weeks	Ergonomics	0	0	0	0	1	**1**
Lidegaard et al. 2018	Denmark; cleaners	Aerobic exercise	12 months	Exercise	0	1	0	0	1	**2**
Linnan et al. 2020	United States; childcare workers	Worksite wellness intervention	6 months	Multicomponent	1	0	0	1	1	**3**
Lippke et al. 2015	Germany; train workers	Computerized lifestyle application to promote multiple health behaviors at the workplace	4 weeks	Multicomponent	1	0	1	1	1	**4**
Lithopoulos et al. 2020	Canada; office workers	Changing Sedentary Behavior in the Office	12 weeks	Exercise	1	0	1	0	1	**3**
Lopes et al. 2023	Portugal; workers	Social robots as health promoting agents	8 weeks	Multicomponent	1	0	1	1	1	**4**

Maddux et al. 2018	Sweden; workers	Yoga	8-16 weeks	Relaxation	1	0	0	0	0	**1**
Mainsbridge et al. 2014	Australia; police and emergency management office workers	E-Health intervention to reduce prolonged occupational sitting	13 weeks	Exercise	0	0	0	1	0	**1**
Mansi et al. 2015	New Zealand; meat processing workers	Pedometer-driven walking program	12 weeks	Exercise	0	0	1	0	1	**2**
Matsugaki et al. 2017	Japan; female nurses	Exercise under the supervision of a physical therapist	12 weeks	Exercise	0	0	0	1	1	**2**
Mayer et al. 2015	United States; firefighters	Worksite exercise program	24 weeks	Exercise	0	1	0	0	0	**1**
Maylor et al. 2018	United Kingdom; office workers	Multicomponent intervention to reduce workplace sedentary behavior	8 weeks	Exercise	0	0	1	1	1	**3**
Mediavilla et al. 2023	Spain; healthcare workers	Doing What Matters in Times of Stress	5 weeks	Mental health	1	1	0	1	1	**4**
Meyn et al. 2022	Germany; office workers	Education-based workplace health promotion program	3 weeks	Diet	1	0	1	1	1	**4**
Miller et al. 2016	United States; university workers	Worksite diabetes prevention intervention	16 weeks	Weight management	1	0	1	1	1	**4**
Milligan-Saville et al. 2017	Australia; managers	"RESPECT" mental health training program	4h	Mental health	1	0	0	1	0	**2**
Milner et al. 2020	Australia, construction workers	Electronic mental health stigma campaign	6 weeks	Mental health	1	0	0	0	1	**2**
Miquel et al. 2023	Germany and United Kingdom; private and public sector workers	"Kelaa" mental resilience app	4 weeks	Mental health	1	0	0	1	1	**3**
Miranda Bispo et al. 2020	Brazil; footwear industy workers	Physical acitivities program	12 months	Exercise	1	0	0	0	1	**2**
Murray et al. 2019	United Kingdom; urban workers	Internet-based physical activity intervention	6-12 months	Exercise	1	0	0	1	1	**3**
Nooijen et al. 2020	Sweden; office workers	Multi-component interventions targeting physical activity or sedentary behaviour	6 months	Exercise	0	1	0	1	1	**3**

Oftedal et al. 2019	Australia; homecare workers	m-Health intervention	4 weeks	Multicomponent	1	0	1	1	1	**4**
Ojo et al. 2024	United Kingdom; office workers	Workplace intervention to reduce sitting	8 weeks	Ergonomics	0	1	1	0	1	**3**
Oka et al. 2019	Japan; nurses	One Stretch	6 months	Exercise	0	0	0	0	1	**1**
Olson et al. 2016	United States; home care workers	COMmunity of Practice And Safety Support	6-12 months	Multicomponent	1	0	1	1	1	**4**
Olson et al. 2016	United States; truck drivers	Safety and health involvement for truckers (SHIFT)	6 months	Weight management	1	0	1	1	1	**4**
Park&Hwang 2024	South Korea; male office workers	Remote physical activity improvement program	12 weeks	Exercise	1	1	1	1	1	**5**
Patel et al. 2016	United States; health insurance workers	Financial incentives to increase physical activity	13-26 weeks	Exercise	0	0	0	0	1	**1**
Patel et al. 2018	United States; workers	Lottery-Based Financial Incentives	6 months	Weight management	1	0	0	0	1	**2**
Pedersen et al. 2019	Norway; logistics workers	Worksite health-promotion intervention	12 months	Exercise	0	0	0	1	1	**2**
Peterman et al. 2019	Australia; office workers	Stand Up Victoria intervention to reduce office workers' sitting time	3 months	Ergonomics	0	1	0	1	1	**3**
Peters et al. 2018	United States; construction workers	Total Worker Health® intervention	6 weeks	Multicomponent	1	1	0	1	1	**4**
Pidd et al. 2018	Australia; manufacturing workers	Workplace alcohol harm reduction intervention	12 months	Addictive behavior	1	1	0	0	1	**3**
Puhkala et al. 2015	Finland; truck and bus drivers	Lifestyle counseling	12 months	Weight management	1	0	1	1	1	**4**
Raedeke & Dlugonski 2017	United States; university workers	Pedometer intervention	10 weeks	Exercise	1	0	1	0	1	**3**
Rameshbabu et al. 2018	United States; custodial workers	Health Yourself: a randomized, tailored self-regulation intervention	6 weeks	Diet	1	0	1	1	1	**4**
Rasotto et al. 2015	Italy; female manufacturing workers	Workplace exercise program for women at risk for neck and upper limb musculoskeletal disorders	6 months	Exercise	0	0	0	1	0	**1**

Reif et al. 2020	United States; university workers	Workplace wellness program "iThrive"	12-24 months	Multicomponent	0	0	0	1	0	**1**
Renaud et al. 2020	Netherlands; office workers	Multi-component dynamic work intervention to reduce sitting time	4-8 months	Exercise	0	0	1	1	1	**3**
Ritngam et al. 2024	Thailand; factory workers	Nurse-led workplace health promotion program	8 weeks	Multicomponent	1	0	0	1	1	**3**
Rollo & Prapavessis 2020	Canada; office workers	Combined health action process approach and mHealth intervention	6 weeks	Ergonomics	0	0	1	1	1	**3**
Roos et al. 2024	Canada; musicians	Injury prevention and wellness program	11 months	Multicomponent	0	1	0	1	1	**3**
Roussel et al. 2015	Belgium; hospital workers	Program for the prevention of low back pain	12 weeks	Multicomponent	1	0	1	1	0	**3**
Sampson et al. 2019	United States; nurses	MINDBODYSTRONG cognitive behavioral skills building program	8 weeks	Multicomponent	1	0	1	0	1	**3**
Santos et al. 2020	Brazil; industrial workers	Progressive restistance exercise program	4 months	Relaxation	1	0	0	0	0	**1**
Sasaki et al. 2021	Vietnam; nurses	Smartphone-based stress management program	10 weeks	Mental health	1	1	0	1	0	**3**
Shrestha et al. 2024	Nepal; hospital workers	Dietary intervention	6 months	Diet	1	0	0	1	0	**2**
Smit et al. 2024	Netherlands; education, assurance, tax, consulting, and retail workers	Integrated workplace health promotion program	6 months	Multicomponent	1	1	0	1	1	**4**
Soler-Font et al. 2019	Spain; nurses	Multifaceted intervention for the prevention and management of musculoskeletal pain	12 months	Multicomponent	1	1	0	1	0	**3**
Suni et al. 2018	Finland; female healthcare workers	Neuromuscular exercise and back care counseling	6 months	Multicomponent	1	0	0	1	1	**3**
Taulaniemi et al. 2019	Finland; female healthcare workers	Neuromuscular exercise	24 weeks	Exercise	0	0	0	1	1	**2**

Taylor et al. 2016	United States; office workers	Booster Break program	6 months	Multicomponent	1	0	0	0	0	**1**
Thiart et al. 2015	Germany; teachers	Internet-based recovery training for sleepless employees with work-related strain	6 weeks	Mental health	1	0	0	1	1	**3**
Thielecke et al. 2024	Germany; farmers	Telephone coaching for the prevention of depression	6-9 months	Mental health	1	0	0	1	0	**2**
Thorndike et al. 2014	United States; physicians	Activity monitor intervention	12 weeks	Multicomponent	0	0	0	1	1	**2**
Thorndike et al. 2016	United States; hospital workers	Incentives to promote employees' healthy food choices	3 months	Diet	1	0	0	1	0	**2**
Ting et al. 2019	Australia; office workers	Workplace-based exercise intervention	12 weeks	Exercise	0	0	0	1	1	**2**
Trombka et al. 2021	Brazil; police officers	Mindfulness-based health promotion	8 weeks	Mental health	1	0	0	0	1	**2**
Umanodan et al. 2014	Japan; manufacturing workers	Computer-based stress management	7 weeks	Mental health	1	1	0	1	0	**3**
van Berkel et al. 2014	Netherlands; research institute workers	Mindful VIP	8 weeks	Mental health	1	0	1	1	1	**4**
van den Brand et al. 2018	Netherlands; workers	Workplace-based group training programme combined with financial incentives on smoking cessation	12 months	Addictive behavior	1	0	0	0	0	**1**
van Holland et al. 2018	Netherlands; meat processing workers	Comprehensive workers' health surveillance program	1-3 years	Multicomponent	0	0	0	1	0	**1**
van Schaaijk et al. 2019	Netherlands; coach drivers	Self-management intervention	7 months	Multicomponent	0	0	1	1	1	**3**
Viester et al. 2018	Netherlands; male construction workers	Worksite intervention for male construction workers	6 months	Weight management	1	0	1	1	1	**4**
Vining et al. 2020	United States; military workers	Chiropractic care	4 weeks	Relaxation	0	0	0	1	1	**2**

Watanabe & Kawakami 2018	Japan; office workers	Multi-component workplace intervention program	3 months	Multicomponent	1	1	0	1	0	**3**
Watanabe et al. 2018	Japan; nurses	Happy Nurse Project	90 days	Diet	0	0	0	0	0	**0**
Watanabe et al. 2019	Japan; nurses	Mindfulness-based Stress Management Program	13 weeks	Mental health	1	0	1	0	1	**3**
Watanabe et al. 2024	Vietnam and Thailand; nurses	Smartphone-based Stress Management Program	7 weeks	Mental health	1	1	0	0	1	**3**
Weinhold et al. 2015	United States; university workers	Diabetes prevention program	16 weeks	Weight management	1	0	1	1	1	**4**
Wilson et al. 2016	Georgia; city/county workers	Diabetes prevention program"Fuel Your Life"	6 months	Weight management	1	0	1	1	1	**4**
Zoellner et al. 2016	United States; workers	Worksite weight loss program	12 months	Weight management	1	0	0	0	1	**2**

SoC = Sense of Coherence, P = Participation, GS = Goal-Setting, V = Variability, E = Empowerment (“1” means that this salutogenic principle is present in the intervention, while “0” means that this salutogenic principle is not present in the intervention; the added numbers equal the “Salutogenesis-Score”)
